# Simultaneous repair of pectus excavatum and congenital heart disease without cardiopulmonary bypass or sternal osteotomy

**DOI:** 10.1186/s13019-014-0168-7

**Published:** 2014-10-16

**Authors:** Yong Sun, Peng Zhu, Shao-Yi Zheng

**Affiliations:** Department of Cardiovascular Surgery, Southern Medical University, GuangZhou, China; Department of Cardiovascular Surgery, Guangdong General Hospital, Guangdong Acedemy of Medical Sciences, 96 Dongchuan Road, Guangzhou, 510080 China

**Keywords:** Pectus excavatum, Congenital heart disease, Minimally invasive surgery

## Abstract

A 8-year-old girl with severe pectus excavatum and an atrial septal defect had simultaneous repair of the both defects, using thransthoracic occlusion for atrial septal defect and improved Nuss technique for the pectus excavatum. Neither cardiopulmonary bypass nor sternotomy was required in this procedure. Details of the procedure and outcome are described.

**Electronic supplementary material:**

The online version of this article (doi:10.1186/s13019-014-0168-7) contains supplementary material, which is available to authorized users.

## Background

Coexisting pectus excavatum and congenital heart disease is not uncommon. Traditionally, the approach to this problem has been to repair each one with a separate surgical procedure because of fear of increased complications from bleeding, infections, and anesthesia [1]. More recently, many reports of successful combined repair have been published [2],[3]. These procedures of pectoralis muscle flaps elevation, resection of the deformed costal cartilages and sternal osteotomy may be associated with increased bleeding because of the cardiopulmonary bypass (CPB) for repair of congenital heart defect. Concomitant repair of a congenital heart disease and pectus deformity is still a challenge. We report a simultaneous repair of an atrial septal defect and pectus excavatum with a technique that combined of thransthoracic occlusion for atrial septal defect and mordified Nuss procedure. Neither CPB nor sternal osteotomy was required in this procedure.

## Case presentation

**Figure 1:**
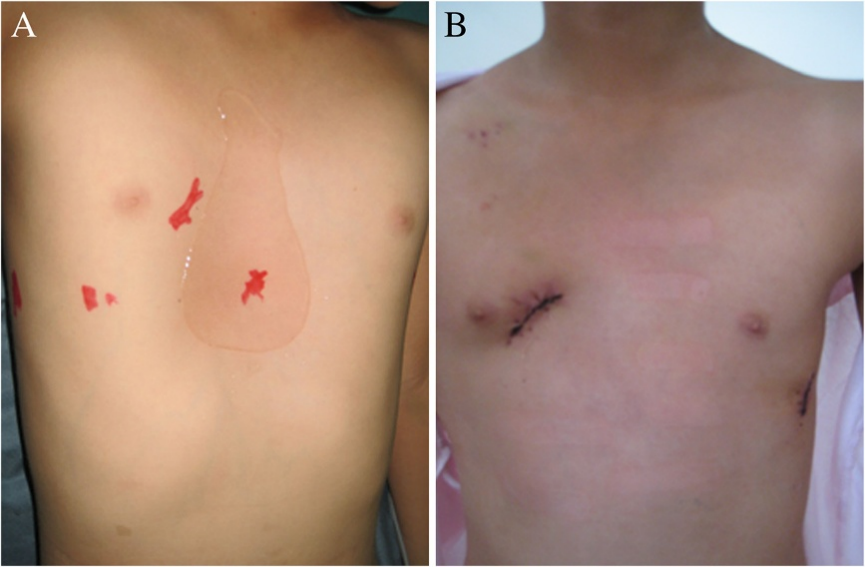
**Patient chest image. (A)** Before cardiac surgery. **(B)** After cardiac surgery.

**Figure 2:**
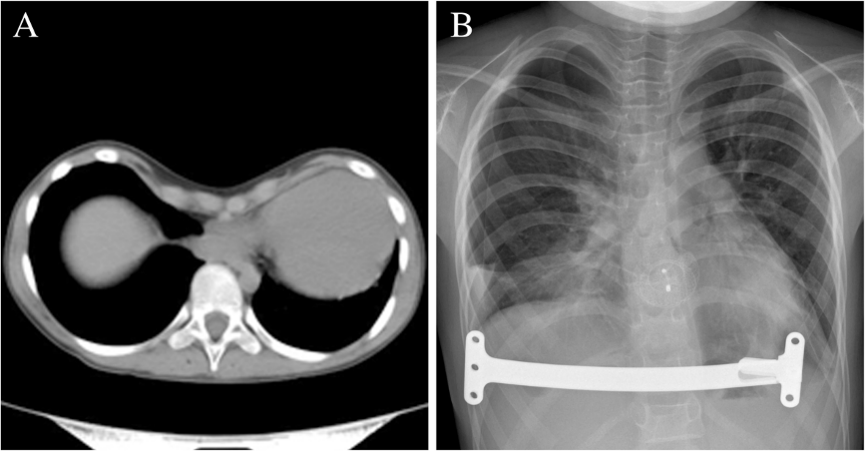
**Imaging of the pectus excavatum. (A)** CT showing the severe asymmetric pectus excavatum with severe cardiac compression and displacement. **(B)** X-ray showing the strutting bar that corrected the funnel chest.

**Figure 3:**
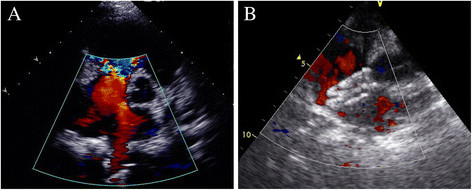
**TEE image showing the atrial septal defect. (A)** Before cardiac surgery. **(B)** After cardiac surgery.

In Jul 2011, an 8-year-old girl was investigated for chest tightness. She had a severe pectus excavatum and atrial septal defect (ASD) (Figure 1A). Computed tomographic scan confirmed the Haller index was 6.2 (Figure 2A) and Echocardiography showed a secundum atrial septal defect with 15 mm size (Figure 3A). Pulmonary function tests were within normal range. Thransthoracic occlusion for atrial septal defect and Nuss technique for the pectus excavatum were considered for Simultaneous repair.

The sites of the operation which included the lowest concaves of the sternum, the highest points of both ribs and the cutting area of the left and right pectus were marked prior to the operation. A 3 cm incision was first made in the patient's right anterior 4th intercostal space. After the pericardium was opened, a 5 mm incision was made in the right atrium for the insertion of the delivery sheath. Under continuous TEE guidance, we used a diameter of 20 mm occluder (Shanghai Memory Alloy Company, Shanghai, China) to close the ASD. The procedure was completed by inspection for residual shunting, atrioventricular valve distortion, or obstruction of the superior vena cava, IVC, or coronary sinus. After the cardiac operation, two 1.5 cm skin incisions were made between the axilla anterior and backward lines on both sides of the pectus wall. With continuous monitoring through the incision for ASD occlusion, an introducing clamp which connected a 18 cm strutting bar (Shanghai Puwei medical instrument Company, Shanghai, China) was slowly moved from the right incision along the collapsed area of the sternum toward the left incision. The convex side of the strutting bar was pulled over the backside of the sternum. When the strutting bar was in the position, the sternum and anterior pectus wall were protruded in an expected shape. After withdrawn the introducing clamp, the strutting bar on one side was invaginated in the holder. Subsequently, the holder and strutting bar were sutured to the rib periosteum. No drainage tube was placed. Postoperative X-ray showed the positions of the strutting bar that corrected the funnel chest (Figure 2B) and the occluder that closed atrial septal defect were satisfactory (Figure 3B).The patient was extubated on the day of surgery and was discharged after 5 days. The cosmetic appearance appeared excellent (Figure 1B). Antiaggregation therapy consisted of heparin (administered during the first 24 postoperative hours) and aspirin (3–4 mg/kg/d, continued for 6 months postoperatively). In Jul 2013, two years after operation, the strutting bar was withdrawn, the cosmetic appearance appeared excellent, there was no episodes of ASD residual fistula, hydrothorax, endocarditis, thromboembolism, or permanent rhythm disturbances.

### Discussion

The combination of cardiac disease and pectus excavatum creates a dilemma for surgeons. A pectus deformity may complicate cardiac surgery by making midline sternotomy technically more difficult. Some authors suggest a two-stage procedure. Jones and colleagues [1] recommend a two-stage approach, correcting the pectus deformity several months before the cardiac pathology. They point out that one-stage procedures may prolong the duration of the operation, may increase the risk of bleeding, may increase sternal infections and may not improve the exposure to the heart. However, others have reported simultaneous repair of both the pectus excavatum and the heart defect achieved good result with no serious complication [2],[3].

The traditional repair of Ravitch has been reported as a means of simultaneous repair of both defects. More recently, the minimal invasive procedure popularized by Nuss were used in these patients with both defects. Okamura and colleagues [4] reported one case, a 47-year-old woman, who had a concomitant pectus excavatum and atrial septal defect repair using a combination of the Ravitch procedure and Nuss procedure, using the convex bar. Kao and colleagues [5] reported a 25-year-old man with severe pectus excavatum and an atrial septal defect had simultaneous repair of the defects, using a patch for closure of the defect and placement of a Nuss bar for the chest wall defect. The results were satisfactory, but all the procedures described above required CPB and sternotomy was not evitable. Our approach for the simultaneous repair for congenital heart defect and pectus excavatum has proven safe and satisfying. Neither CPB nor sternotomy was required in this procedure.

## Conclusions

Simultaneous repair of an atrial septal defect and pectus excavatum with the techniques that combined of thransthoracic occlusion for atrial septal defect and mordified Nuss procedure is simple, minimal invasive and feasible.

## Consent

Written informed consent was obtained from the patient's guardian for the publication of this report and any accompanying images. A copy of the written consent is available for review by the Editor-in-Chief of this journal.

## Authors' contributions

YS performed the operation and drafted the manuscript. PZ collected the clinical data and participated the operation. SYZ designed the operation, revised and submitted the paper. All authors read and approved the final manuscript.

## Authors' information

YS and PZ are surgeons of Department of cardiovascular surgery, Guangdong General Hospital, Guangdong Acedemy of Medical Sciences and postgraduate students of Department of Cardiovascular Surgery, Southern Medical University. SYZ is the chief surgeon of Department of cardiovascular surgery, Guangdong General Hospital, Guangdong Acedemy of Medical Sciences.

## Authors’ original submitted files for images

Below are the links to the authors’ original submitted files for images. Authors’ original file for figure 1 Authors’ original file for figure 2 Authors’ original file for figure 3

## Abbreviations

CPB: Cardiopulmonary bypass
ASD: Atrial septal defect
TEE: Transesophageal echocardiography
CT: Computed tomography
