# Supplementary figures and images for: Simultaneous repair of pectus excavatum and congenital heart disease without cardiopulmonary bypass or sternal osteotomy
Source: J Cardiothorac Surg. 2014 Oct 16;9:168. doi: 10.1186/s13019-014-0168-7 (PMC4203894; doi:10.1186/s13019-014-0168-7)

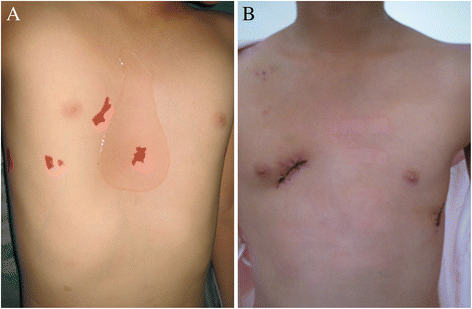

Supplement: Supplementary file 1 — Authors’ original file for figure 1 [file 13019_2014_168_MOESM1_ESM.gif]

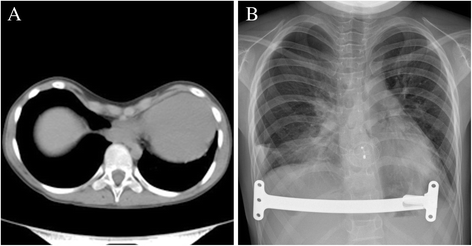

Supplement: Supplementary file 2 — Authors’ original file for figure 2 [file 13019_2014_168_MOESM2_ESM.gif]

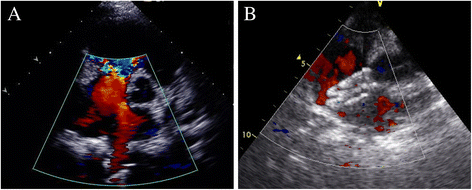

Supplement: Supplementary file 3 — Authors’ original file for figure 3 [file 13019_2014_168_MOESM3_ESM.gif]
